# A survey on personnel awareness of the factors affecting accurate blood pressure measurement in the medical centres of Jahrom County

**DOI:** 10.1002/nop2.403

**Published:** 2020-04-16

**Authors:** Safar Zarei, Fatemeh Nasimi, Hassanali Abedi, Najmeh Sadeghi

**Affiliations:** ^1^ Department of Physiology Faculty of Medicine Jahrom University of Medical Sciences Jahrom Iran; ^2^ Department of Intensive Neonatal Care Nursing Faculty of Nursing Jahrom University of Medical Sciences Jahrom Iran; ^3^ Research Center for Non‐Communicable Diseases Jahrom University of Medical Sciences Jahrom Iran; ^4^ Sirjan School of Medical Sciences Sirjan Iran

**Keywords:** assessment, awareness, blood pressure, clinical skill, environmental exposure, nursing

## Abstract

**Aims and objectives:**

The main purpose of this study was to determine the clinical skill of the medical personnel on level of awareness of standard methods used during blood pressure measurement.

**Background:**

Blood pressure measurement is one of the vital clinical proficiencies the hospital personnel must be equipped with. Results from different surveys highlight the importance of awareness amongst medical personnel in controlling blood pressure.

**Design:**

Descriptive cross‐sectional study.

**Methods:**

Using standardized questionnaires devised by the researcher, data were collected from 302 participants working in healthcare centres in Jahrom. The extracted data were analysed using SPSS.

**Results:**

Observations showed that 10–20% of the participants had wide knowledge of influential factors affecting blood pressure measurement. Moreover, there was a meaningful relation between holding higher degrees and accurate blood pressure measurement (*p* < .05). Nevertheless, besides the personnel holding lower degrees, those holding higher educational degrees also had dearth of knowledge of factors affecting blood pressure measurement.

**Conclusions:**

The overall findings of this study indicate that the knowledge among the hospital personnel in determining factors affecting blood pressure measurement was inadequate.

## 1 INTRODUCTION

Hypertension is one of the most widely prevalent diseases worldwide and is considered as one of the leading causes of global morbidities and premature mortalities (Mills et al., 2016). With the crude prevalence of 22%, its rate is increasing not only in Iran but also worldwide (Mirzaei, Moayedallaie, Jabbari, & Mohammadi, 2016).

The recently updated American College of Cardiology/American Heart Association (ACC/AHA) 2017 guidelines have been modified to diagnose any patient with a systolic blood pressure ≥130 mm Hg as hypertensive in place of earlier guidelines, which had a cut‐off value of ≥140 mm Hg. ACC/AHA 2017 guidelines categorize blood pressure (BP) as normal, elevated and hypertension (stages 1 and 2) based on ≥2 measurements on ≥2 separate occasions. Hypertension is defined as BP reading of at least 130 mm Hg systolic and 80 mm Hg diastolic with the corresponding 24‐hr ambulatory BP readings being 125 mm Hg systolic and 75 mm Hg diastolic (Whelton, 2018). Hypertension is one of the major risk factors for coronary artery disease, stroke, myocardial infarction, heart failure and long‐term kidney disease. In addition, it is a modifiable risk factor with non‐pharmacological and pharmacological measures providing substantial risk reduction of these conditions (Turner, Viera, & Shimbo, 2015). In clinical practice, accurate blood pressure measurement (ABPM) has diagnostic, prognostic and therapeutic utility. ABPM is considered the gold standard in diagnosing hypertension, including white coat, masked and nocturnal hypertension (Parati et al., 2014). BP can be measured by both direct and indirect methods, of which the indirect method is more prevalent in hospitals nowadays. As most hypertensive patients need lifelong treatment, accurate diagnosis of factors contributing to high BP is a necessity.

Errors committed during regular BP measurements might be due to the lack of knowledge of environmental factors influencing BP or the personnel being inattentive to those factors. Jahrom, a city in the south‐west of Fars Province in Iran, has a medical university that also caters the medical needs of patients from neighbouring counties. Thus, investigating the existing shortcomings in the awareness and knowledge of medical science experts can influence the scheduling methods and finally can help to solve the problems in Jahrom hospitals and finally improve the quality of medical education among students (Zarei, Bigizadeh, Pourahmadi, & Ghobadifar, 2012).

## 2 BACKGROUND

When interpreting BP measurement, there could be sharp differences in readings among different measurers and such differences could probably be due to insensitivity to details of temperature, busy and noisy medical environment, patient's condition concerning the stress, etc. Some studies have noted deficient knowledge of ABPM skills in nursing students and clinical nurses (Gillespie & Curzio, 1998; Thavarajah, White, & Mansoor, 2003). Besides, hearing impairment is one of the potential causes of error; hence, medical society must pay proper attention to the age and hearing threshold of the tester. A study showed that 33% of physicians in London had not passed formal education courses in BP measurement techniques (Feher, Harris‐St John, & Lant, 1992). Another investigation performed at a hospital in the United States showed that only 30% of personnel were able to diagnose BP changes of 2 mm Hg (Kemp, Foster, & McKinlay, 1994). Additionally, negligence of accurate readings and interpreting principles including repeating previous readings and terminal digit and single‐number preference result in inaccuracy and biased results (Wingfield et al., 2002).

The above points affect the prognosis of hypertension and can result in medication errors. A Montreal‐based study indicated that 78% of BP readings were rounded to an integer (Wen, Kramer, Hoey, Hanley, & Usher, 1993), resulting in observer error.

Many factors affect inaccurate BP measurement, including errors in the method of measurement (unsuitable size of cuff), unsuitable and unsupported hand position, applying the cuff inaccurately, having problem in hearing Korotkoff sounds, smoking, stress, anxiety and fear of white coats (Kario, 2010). Other related studies in literature have shown the importance of adhering to best clinical practices while measuring BP, which include calming the patient down, keeping the environment silent and ensuring proper assessment when the patient has pain. Neglecting these factors can result in overestimation of BP interpretations (Anderson & Maloney, 1994; Shapiro et al., 1996).

### 2.1 Aim

Concerning the importance of BP and the measurement procedure applied, environmental factors and accurate BP readings affect accurate measurement. Hence, different countries have embarked on different economics of patient safety to reduce such errors as well as to enhance the quality of their healthcare services.

## 3 METHODS

### 3.1 Study design

Initially, study sample included 400 personnel working in healthcare centres in Jahrom. However, participant dropout left us only with 302 members—134 nursing aides, 123 nurses and 45 physicians. Nearly, half of the nursing aides were between 20‐30 years old and most of the physicians were between 30‐40 years old. Sampling was done randomly.

#### 3.1.1 Setting and sample

**Figure 1:**
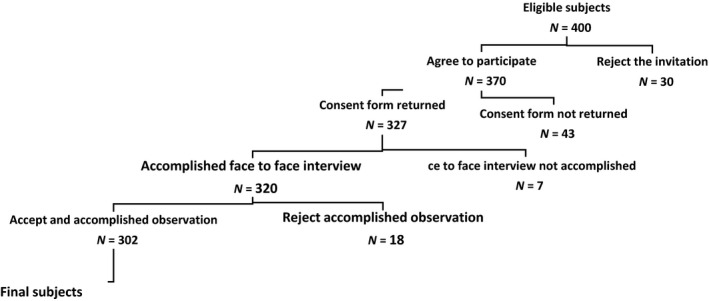
Study participant status

To extract the due information about the extent of knowledge and awareness of personnel involved in measuring patients' BP, patient consent was first obtained. While the personnel were busy measuring the BP, the observation was performed (Figure 1).

#### 3.1.2 Ethical considerations

The present study was performed pursuant to the medical ethics stipulated by the Ministry of Health, Treatment and Medical Education of the Islamic Republic of Iran. Prior informed consent was obtained from all participants. The ethics committee of Jahrom University approved this study (JUMS‐85).

#### 3.1.3 Data collection

The instruments for data collection in this study were 10‐min interviews, direct observation of the methods of BP measurement and filling in the standardized questionnaire prepared by the researcher containing all the basic and necessary information about factors affecting ABPM including drinking tea/coffee recently, recent physical activity, having smoked recently, hand level, room temperature, sitting position on the chair, knowledge about the timing of and resting before the BP measurement, room being quiet, patient talk, nurse talk, patient pain and hearing impairment. The knowledge of nurses, nursing aides and physicians was investigated as follows.

In the questionnaire, questions with “yes‐no” responses for each item were included. If the respondent answered “yes,” it indicated that the participant possessed enough knowledge about that item. To confirm the findings, three or four items were asked and positive answers showed that the participant had correct knowledge about the issue. An answer was considered correct if it met the criteria listed in the *Fundamentals of Nursing* by Potter and Perry [8]. The researcher then observed the method of BP measurement used by personnel directly without any guidance. The participants were considered as having enough theoretical knowledge about the procedure when measurements were performed in accordance with standard practice. However, in some cases, the participants ignored certain rules essential or central to the nature of correct measurement.

At the end of the experiment, a 10‐min interview was conducted with personnel to record their comments and interpretations on their failure to take BP measurements properly. They were also requested, in a blinded manner, to provide suggestions or solutions to overcome the failure.

#### 3.1.4 Statistical analysis

Professional expertise was used to verify the content validity of the questionnaire. Its reliability was tested through 20 questionnaires filled in by 20 volunteers. Cronbach's alpha revealed a 77% admissible rate. The extracted data were analysed using SPSS statistical software (version 16) and explained using descriptive statistics and cross‐tabulations. The chi‐squared test was used to compare the averages. Multinomial logistic regression was conducted to compare the relationship between the data obtained from the questionnaire and the observation. The level *p* < .05 was considered statistically significant, and the results were reported in percentage values.

## 4 RESULTS

Given that this study was conducted on a wide range of personnel including nurses, physicians, nursing aides and each with a different level of education, it was clear that there was a meaningful relation between holding a higher educational degree and ABPM; namely, the higher the educational degree, the more accurate the BP measurement (Table 1). About the hearing level, just 2% of the sample cases were afflicted with hearing impairments and had difficulty in recognizing Korotkoff sounds. Only 2.2% of physicians, 0.9% nurses and none of the nursing aides were cognizant of the impact of hearing impairments on ABPM (Fisher's exact test: 6.02, *p* = .1) and the reason can be defects in pre‐employment examinations. Concerning the necessity of taking a rest before the measuring procedure, 25% of physicians, 14% of the nurses and 16.7 of nursing aides answered the questionnaire correctly and knew that it is mandatory for the patient to take a 5‐min rest before the measurement. However, 25% of physicians, 13.2% of nurses and 14% of nursing aides performed it during the observation while some others performed the procedure with wrong rest time (Fisher's exact test: 8.4, *p* = .5). The reason could be insufficient exposure of personnel to necessary information during their university education.

**Table 1:** Accurate knowledge of blood pressure measurement based on educational degrees

Physician	Nurse	Nursing aide	*p* value
31.6%	11%	9.4%	.018

Concerning patient's talk, 47.50% of the physicians, 16.7% of nurses and 13.8% of the nursing aides knew that when the patient is talking, the first thing to do is to keep him/her silent and then measure the BP. However, our observation showed that just 37.5% of the physicians, 16.1% of the nurses and none of the nursing aides were mindful of it (Fisher's exact test: 3.4, *p* = .2). The reason could be problems in remembering such points and even forgetting them after graduation.

Regarding patient smoking patterns, 47.50% of the physicians, 7% of nurses and 8.7% of the nursing aides knew that they should ask the patient about smoking before the measurement and if the patient has smoked, they must delay the measurement for 30 min. Also, our observation showed that 15.4% of physicians, 7.9% of nurses and 8% of the nursing aides were mindful of patient’s “not smoking” status for the measurement (Fisher's exact test: 6.08, *p* = .4; Table 1). When asking the patient whether he/she has drunk tea or coffee before the measurement, only 12.5% of the physicians, 4.4% of nurses and 3.6% of nursing aides answered correctly and knew this matter while none of them paid attention to it (Fisher's exact test: 4.062, *p* = .4).

Regarding room temperature, only 15% of the physician, 7% of nurses and 3.6% of nursing aides knew that room temperature must be between 25 and 30°C. During the observation, just 10% of physicians, 2.50% of the nurses and none of the nursing aides paid attention to it (Fisher's exact test: 3.6, *p* = .5). The reason could be that they were naïve.

Considering the patient's pain before measuring the BP, 40% of physicians, 20.2% of nurses and approximately 13% of the nursing aides were aware of the matter while observations showed that 50% of physicians, 2.5% of nurses and none of the nursing aides were mindful of it (Fisher's exact test: 3.6, *p* = .5). The reason could be that these personnel had not improved and updated their knowledge.

As regards the sitting position of the patient on the chair, 10% of physicians, 13.2% of nurses and 7.2% of nursing aides knew that the patient must be rested on the chair and legs should be uncrossed while observations showed that 65% of the physicians and 80% of the cases in the other two groups paid no attention to the impact of patient's position on BP measurement.

About the length of time the patient must sit in a chair before measuring the BP, 70% of the cases in all three groups were not aware of this time frame and observations showed that 15% of the physicians, 13% of nurses and none of the nursing aides were mindful of it (Fisher's exact test: 5.02, *p* = .08). The reason could be lack of enough study and shortage of workshops to update their knowledge.

With regard to keeping the environment silent, only 30% of the physicians, 17.5% of nurses and 13% of nursing aides were aware of this aspect.

As far as patient anxiety before the procedure is concerned, just 32.5% of physicians, 15.8% of nurses and 8.7% of nursing aides knew that the patient should not be anxious. However, observations showed that 12% of the physicians, 10% of nurses and none of the nursing aides were mindful of it (Fisher's exact test: 1.5, *p* = .6). About elbow position, 60% of physicians and around 20% of nurses and nursing aides were aware of the right position while observations showed that none of them were mindful of it.

Regarding cuff placement, 95.6% of physicians, 86.2% of nurses and 92.5% of nursing aides knew that they should not fasten the cuff on the clothes while only 50% of physicians and 33% of subjects in the other two groups adhered to this during observation (Fisher's exact test: 1.5, *p* = .7). Reasons included that workload did not permit them to study and review these points and perform the measurement correctly.

With respect to fist position, 32% of physicians and 15% of the cases in the other two groups knew that the patient's hand must be unfisted and the fingers must be extended while 50% of physicians, 33% of nurses and 16.7% of nursing aides were mindful of it (Fisher's exact test: 1.3, *p* = .6). The reason for such negligence could be due to time scarcity and the lack of knowledge improvement of personnel following their graduation from university (Table 2).

**Table 2:** Healthcare personnel awareness of accurate blood pressure measurement and its influential factors

Title	Mode of data collection	Nurse assistance *N* = 138	Nurse *N* = 114	Physician *N* = 40	*p* value	Correlation
1. Drinking tea/coffee recently	Questionnaire	3.6%	4.4%	12.5%	.0001	*R* = −1
	Observation	–	–	5.4%		
2. Recent physical activity	Questionnaire	9.4%	15.8%	22.5%	.0001	*R* = 1
	Observation	3.1%	4.5%	6.2%		
3. Having smoked recently	Questionnaire	8.7%	7%	47.5%	.0001	*R* = −1
	Observation	8%	7.9%	15.4%		
4. Arm should be the same level as heart	Questionnaire	26.8%	37.7%	92.5%	.0001	*R* = 1
	Observation	8.2%	20%	60%		
5. Room temperature should be 20–25°C	Questionnaire	3.6%	7%	15%	.0001	*R* = 1
	Observation	–	2.5%	10%		
6. Knowledge about the time and position of sitting on the chair	Questionnaire	7.2%	13.2%	10%	.0001	*R* = 1
	Observation	–	13%	15%		
7. Resting before blood pressure measurement	Questionnaire	16.7%	13.2%	25%	.0001	*R* = −1
	Observation	14%	14%	25%		
8. Room should be quiet	Questionnaire	13%	17.5%	30%	.069	*R* = 1
	Observation	–	1.4%	2.5%		
9. Patient shouldn't talk	Questionnaire	13.8%	16.7%	47.5%	.0001	*R* = −1
	Observation	–	16.1%	37.5%		
10. Nurse shouldn't talk with the patient	Questionnaire	2.9%	2.6%	2.5%	.0001	*R* = 1
	Observation	–	–	–		
11. Patient has pain or not	Questionnaire	13%	20.2%	40%	.0001	*R* = −1
	Observation	–	2.5%	50%		
12. Knowledge about the effect of hearing impairment on blood pressure measurement	Questionnaire	2.2%	0.9%	0%	.202	*R* = 1
	Observation	–	–	–		
13. Knowledge of referral to physician for the personnel having hearing deficit by boss	Questionnaire	13.7%	39.2%	47.1	.571	*R* = −1
	Observation	–	–	–		
14. Knowledge of cross leg when sitting on the chair	Questionnaire	13%	13.2%	20%	.084	*R* = −1
	Observation	10%	10%	15%		
15. Knowledge of effect of patient anxiety on blood pressure	Questionnaire	8.7%	15.8%	32.5%	<.001	*R* = −1
	Observation	‐	10%	12%		
16. Knowledge of placing the cuff on arm without clothing	Questionnaire	95.6%	86.2%	92.5%	.187	*R* = 1
	Observation	33%	33.3%	50%		
17. Knowledge that the fingers shouldn't fist	Questionnaire	16.7%	33%	50%	.114	*R* = −1
	Observation	15%	15%	32%		
18. Knowledge of providing support under the arm	Questionnaire	4%	5%	5%	.94	*R* = 1
	Observation	1.4%	2.5%	2.6%		

Regarding keeping the hand at the same level as the heart, just 92.5% of physicians, 37.7% of nurses and 26.8% of the nursing aides were aware of it. However, our observations indicated that only 60% of the physicians, 20% of the nurses and 8.2% of the nursing aides paid attention to this rule and measured the BP correctly and kept the hand at the same level as the heart (Fisher's exact test: 98.7, *p* < .001). The reason, as stated by the subjects, could be the increasing number of visitors referring daily to these healthcare centres to receive cares.

The systolic and diastolic differences of pressure on rest and unrest state of hand are shown in Table 3. Only 4.1% of nursing aides and 5.2% of physicians and nurses knew to make a support for the hand. However, observations showed that 2.6% of physicians, 2.5% of nurses and 1.4% of nursing aides followed this rule and provided a suitable support for the hand while measuring the BP (Table 3). In relation to all the aforementioned variables, there was a positive correlation between questionnaire and observation results (*R* = 1).

**Table 3:** Systolic and diastolic blood pressure, hand in rest and unrest positions, and heart at even level to the hand

Blood pressure	Systole mm Hg	Diastole mm Hg
Hand supported and at the same level as the heart	125	82
Hands is unsupported	127	91

## 5 DISCUSSION

The present study investigated the knowledge of healthcare centre personnel concerning different factors affecting ABPM and was performed on three sample groups of physicians, nurses and nursing aides. The study is important as the procedure adopted is one of the most prevalent techniques to control and diagnose hypertension, cardiovascular diseases, hypotension and brain attacks from various external factors. Previous results certified that nearly 80% of nurses prior to filling the forms thought that they had enough command of (theoretical and practical) knowledge about factors affecting BP measurement. However, despite their daily involvement, only 30% scored above 60 in the test (da Costa Farias Almeida & Lamas, 2013).

Another study showed that nearly 12% of the cases under the study had hearing impairments; hence they were unable to thoroughly diagnose systolic and diastolic pressure points.

Hearing impairment obtrudes hypertension diagnosis and causes fatal errors. Hearing impairment causes pseudo‐high diastolic pressure and pseudo‐low systolic pressure. Nurses must not suffer from hearing and visual impairments and must be able to observe the mercury pillar without any tension in their body organs (Pickering et al., 2005). In the present study, 25% of the physicians were aware of the importance of patient rest before performing the procedure and among them, 5% were not cognizant of the recommended time lapse for measuring BP. Previous studies have shown that previous readings can usually affect the followings; hence, considering the above‐mentioned points can help in obtaining accurate BP readings (Gillespie & Curzio, 1998). In a study in Tehran, nearly 30% of the personnel did not calm down the patients prior to measuring the BP (Anderson & Maloney, 1994). Therefore, preparing an exact and updated criterion based on predefined standards is suggested to prevent shortcomings in the techniques of measurement. Besides, thorough supervision is needed in order for all personnel to study and make use of them.

Prior to measuring BP, patients' conditions including rest time, physical activity, anxiety and smoking are of high importance. Previous findings proved that negligence of the above‐mentioned cases may cause pseudo hypertension up to 30 mm Hg. Therefore, a time lapse of at least 30 min is recommended before measuring the BP (Low et al., 1995; White, 2007).

In a hospital‐based study in Tehran, almost 70% of nurses were aware of the above‐mentioned standards prior to performing the procedure (Anderson & Maloney, 1994). Similarly, nearly 11% of nurses and nursing aides were aware but paid no attention.

The correlation between completing the questionnaire and observation did not show a positive relationship. We noticed that a high percentage of personnel neither had enough knowledge about the procedure nor performed it correctly.

Being mindful of the patient's position while measuring the BP is of vital importance. Sitting position on the chair involves leaning back, uncrossing the legs for 2 min and calming down the patient when measuring the BP. The present study indicated that around 10% of the cases under the study in all the three groups were cognizant of the above‐mentioned points and 80% of the cases were knowledgeable about the effect of the patient's position on causing hypertension. Based on previous findings, the cross‐legged position may cause hypertension (Pickering et al., 2005). Furthermore, findings have proved that noisy environment, patient talking to the nurse or vice versa may cause pseudo hypertension (Pickering et al., 2005; Torrance & Serginson, 1996). Talking results in a high number of heart beats and increases both systolic and diastolic pressures (Bosetti, Turati, & La Vecchia, 2014). Our investigation asserts that a higher level of awareness is related to a higher level of education. This necessitates holding technical courses in practical methods for personnel with lower degrees by healthcare and hygiene bodies. In a study performed on nursing students in Madrid, nearly 60% of the students were aware of the standard leg position and 90% of them were knowledgeable about the recommended hand position at the same level as the heart (Gonzalez‐Lopez et al., 2009). As to locating the hand and heart evenly, most of the students followed the recommended instructions. In a study performed on nursing students in the USA, 18% of the cases knew the importance of providing support for the hand when measuring BP (Torrance & Serginson, 1996). According to the provisions of the American Heart Association (AHA), the hand must be leaned over during the BP measurement. In cases where the hand is not leaned over on the table and is left without any support, isometric contraction occurs and when the hand is placed above or under the heart level, diastolic pressure either increases or decreases by 10% due to the effects of hydrostatic pressure (this has been approved in the present study; Pickering et al., 2005; Torrance & Serginson, 1996). There was a positive correlation between knowledge and correct measurement among the above‐mentioned variables. However, some of the nurses with knowledge of this procedure did not adhere to AHA standard guidelines.

### 5.1 Observations

The most common mistakes made by physicians, nurses and nursing aides were as follows: measuring BP with the patient in the sitting position without leaning his/her back; hand not supported and is stretched and is at a lower level to the heart; measuring BP with the patient in the supine position at other times, etc. A previous study showed that diastolic pressure in the sitting position is 5% lower than in the supine position (Bailey & Bauer, 1993). Care must be taken to ensure that the mercury manometer is in a vertical state when measuring the BP. However, we observed that some of the participants neglected this aspect and the mercury column was tilted. Furthermore, around 10% of the cases applied the cuff and the stethoscope on the patient's clothing and only in less than 10% of the cases, the patient's clothing was tied upwards, which resulted in agitation of sympathetic nerves and hypertension (de Greeff et al., 2010).

### 5.2 Limitations

Study limitations include limited funding and facilities, use of aneroid manometers rather than mercury and digital manometers, and personnel and patient dropout. Furthermore, some of personnel did not fill the questionnaire as desired. Although some of the participants had enough knowledge, they stated that they were too busy and did not have enough time to adhere to the standard guidelines.

## 6 CONCLUSIONS

This study proved that some personnel, despite having good knowledge, neglected rules and standards because of lack of follow‐up measures and decreasing knowledge with increasing work time. Other causes of negligence were the high workload and long work hours as well the disproportionate number of personnel compared to the number of patients. Also, some environmental factors like temperature, and a noisy and busy environment affect the results of BP measurements.

## 7 SUGGESTIONS

Blood pressure measurement procedure is one of the most prevalent techniques for diagnosing hypertension, cardiovascular diseases, hypotension and brain attacks from different external factors. In order to expand the knowledge of personnel on the influential environmental factors on ABPM, hospital managers must set up scheduled and regular programmes in the form of workshops and assess the personnel's knowledge and practical abilities after the end of that workshop.

## 8 RELEVANCE TO CLINICAL PRACTICE

The findings of the present study indicate the low command of knowledge among healthcare centre personnel in determining influential factors on BP measurement as this poor knowledge can lead to misdiagnosis of hypertension and hypotension.
