# Corrigendum

**DOI:** 10.1002/nop2.594

**Published:** 2020-08-03

In Zarei *et al*. (2020), the author affiliations listed for Najmeh Sadeghi were incorrect. Najmeh Sadeghi is only affiliated to ^4^Sirjan School of Medical Sciences, Sirjan, Iran. Therefore, the correct details for this article are as follows:

Safar Zarei^1^, Fatemeh Nasimi^2^, Hassanali Abedi^3^, Najmeh Sadeghi^4^

^1^Department of Physiology, Faculty of Medicine, Jahrom University of Medical Sciences, Jahrom, Iran

^2^Department of Intensive Neonatal Care Nursing, Faculty of Nursing, Jahrom University of Medical Sciences, Jahrom, Iran

^3^Research Center for Non‐Communicable Diseases, Jahrom University of Medical Sciences, Jahrom, Iran

^4^Sirjan School of Medical Sciences, Sirjan, Iran

The authors apologize for this error.
